# Rapid early innate control of hepatitis C virus during IFN-α treatment compromises adaptive CD4^+^ T-cell immunity

**DOI:** 10.1002/eji.201142072

**Published:** 2012-08-06

**Authors:** Tom Pembroke, Ian Rees, Kathleen Gallagher, Emma Jones, Paul Mizen, Timur Navruzov, Andrew Freedman, Ceri Fielding, Ian R Humphreys, Eddie C Y Wang, Awen M Gallimore, Andrew Godkin

**Affiliations:** School of Medicine, Institute of Infection and Immunity, Cardiff University, TheHenry Wellcome BuildingCardiff, UK

**Keywords:** CD4^+^ T cells, Hepatitis C virus, IFN-α, NK cells

## Abstract

The ability to control HCV with IFN-α-based treatments provides an opportunity in humans to study how the rate of viral clearance in vivo impinges on the development of antiviral responses. Ex vivo (IFN-γ-producing) and cultured antiviral CD4^+^ T cells, serum cytokines, and viral loads were measured repeatedly in a cohort of chronically HCV-infected subjects (*n* = 33) receiving IFN-α. Rapid control of virus indicated by an increased calculated rate of virus clearance, occurred in those subjects demonstrating absent/minimal T-cell responses (*p* < 0.0006). Surprisingly, in subjects who demonstrated the most robust T-cell responses (and reduced serum IL-10), there was actually a reduced rate of early virus clearance. A subsequent analysis of NK-cell function in available subjects (*n* = 8) revealed an inverse correlation between pretreatment NK-cell expression of NKp46 and the potential to upregulate cytotoxic function on exposure to IFN-α (*p* < 0.004), as well as the subsequent measured rate of viral clearance (*p* = 0.045). Thus, the CD4^+^ T-cell response during IFN-α treatment appears to be shaped by the rate of innate virus suppression. These data suggest that individuals who respond most effectively to immune intervention may be most in need of subsequent vaccination to prevent reinfection.

## Introduction

Hepatitis C virus (HCV) causes a chronic infection with the potentially serious sequelae of cirrhosis and liver cell cancer. Although a small virus of 9 kb, several of the encoded proteins have roles in counteracting the effects of type-I IFNs (IFN-α/β) and their signaling pathway (reviewed in 1) supporting its fundamental role in containing and/or eliminating the virus. Current treatment centers on giving exogenous IFN-α although the exact mechanism of viral clearance in treated subjects remains unclear. The ability to control and eradicate HCV with treatment makes it unique among most human chronic viral infections.

CD4^+^ T cells appear to play a fundamental role in controlling noncytopathic virus infections such as HCV, hepatitis B virus, HIV, and lymphocytic choriomengitis virus (LCMV; reviewed in [2]). The importance of adaptive CD4^+^ T-cell responses in clearing HCV is underpinned by the observation that spontaneous clearance of the virus has been associated with particular HLA-DR antigens [3]. We and others have previously observed that subjects who have cleared the virus demonstrate more robust antiviral CD4^+^ T-cell responses than chronically viremic subjects [4–9]. The majority of individuals infected with HCV develop chronic infection, and these patients rely on IFN-α-based treatment to eradicate the virus. IFN-α/β has a complex interaction with T cells (reviewed in [10]). There is some evidence that IFN-α may influence the priming and differentiation of naïve human lymphocytes, encouraging Th1-type IFN-γ-producing cells to develop [11]. We have recently shown that IFN-α can also impinge on certain memory subsets of human CD4^+^ T cells [12]. It has been assumed that not only do antiviral CD4^+^ T cells play a role in clearing the virus in patients undergoing treatment, but that the measurement of these CD4^+^ T-cell responses is a useful indicator of therapeutic outcome [13].

IFN-α can also activate innate immune cells such as NK cells. NK cells compose approximately 10–15% of peripheral blood lymphocytes but are enriched in the intrahepatic compartment suggesting they are well placed to impinge on HCV. The signaling required to activate NK cells is a balance between activating receptors (e.g. NKG2D, NKp30, and NKp46) and inhibitory receptors (e.g., killer cell immunoglobulin like receptors (KIRs), NKG2A). The rapid pathogen-induced response of NK cells includes direct perforin-mediated cytotoxicity and cytokine release [14]. Polymorphisms in HLA type and KIR molecules on NK cells that result in diminished inhibitory signaling are associated with protection against HCV [15]. Additional studies have suggested a reduced NK-cell function and frequency in chronic HCV infection, which reverses with treatment [16–18]. Furthermore, there is evidence that superior NK-cell responses identify subjects who have been exposed to HCV yet remain uninfected [19].

There is much interest currently in understanding how innate responses control adaptive immune responses and vice versa. The ability to control and eradicate HCV with treatment allowed us to examine aspects of this relationship. In this study we primarily explored how the rate of control of viremia, which impacts on antigen availability, impinges on the development of antiviral CD4^+^ T-cell responses. For this purpose, we performed frequent serial measurements of a range of parameters during treatment including CD4^+^ T-cell responses; serum cytokine levels; liver enzymes; and viral loads along with pretreatment NK-cell function. Results of these analyses enabled us to probe the nature of the relationship between the innate and adaptive immune system and furthermore, to examine factors that might contribute to generating a virus-specific CD4^+^ T-cell immune response and/or successful outcome during treatment.

## Results

### Demographic and clinical details of patients: viral load and ALT predicted treatment outcome

Sustained virologic response (SVR), or treatment success, is defined as a negative viral PCR at both the end of treatment and at 6 months following the completion of treatment. Treatment failure is defined by a positive viral PCR at the end of treatment and/or 6 months following completion. Prior to commencing treatment, chronicity of infection in this cohort was established by the presence of HCV-specific antibody and two positive viral PCR at least 6 months apart.

Thirty-three patients chronically infected with HCV were studied in detail at multiple time points (viral loads and alanine aminotransferase (ALT) levels detailed in Table 1). Of these 33 patients, 24 demonstrated a successful eventual treatment outcome with an SVR at 12–18 months. An ALT < 45 IU/ml (the upper limit of normal range as commonly accepted and measured by the biochemistry laboratory University Hospital of Wales) combined with a drop in viral load to <1000 IU/ml significantly identified patients at day 28 who went on to eradicate the virus and gain an SVR (*p* = 0.00013 Fisher’s exact test; predicted treatment success 100% specificity, 75% sensitivity). Reduced viral load by day 28 has been identified previously as a factor associated with eventual treatment success [20,21]. Although baseline high viral load has previously been described as an indicator of treatment failure [22], no such correlation was observed in these patients (Table 1).

**Table 1:** The characteristics of HCV-infected patients who were studied immunologicallya)

Patient	Age	Gender	HCV genotype	Fibrosis score	ALT day 0	ALT day 28	Viral load day 0	Viral load day 28	Treatment duration
Group 1: treatment failure									
1A	54	m	3	nd	103	49	2,070,684	86	6
1B	53	m	3	4	165	85	514,722	0	6
1C	43	m	1	6	312	163	761,046	70,776	12
1D	50	f	1	6	117	99	3,265,796	665,747	3
1E	58	m	3	2	72	37	1,011,100	288,632	6
1F	54	m	2	4	194	240	4,078,688	156	6
1G	58	m	3	6	53	32	308,268	25,064	5
1H	51	m	1/3	1	39	22	3,763,573	19,2710	3
1I	29	f	4	nd	38	22	620,568	17,3316	3
Group 2: treatment success (SVR) with absent/ transient weak CD4^+^ responses									
2A	44	m	3	1	58	26	2,568,800	0	6
2B	44	m	3	0	76	35	61,816	0	6
2C	38	m	1	1	460	45	123,797	0	12
2D	43	m	3	1	77	42	787,310	100	6
2E	41	f	3	1	101	41	10,240,942	0	6
2F	35	m	3	nd	103	113	57,298	0	6
2G	47	m	3	nd	169	43	4,744,286	0	6
2H	58	m	1	2	72	37	24,478	0	12
2I	38	m	3	1	23	24	1,618,700	0	6
2J	55	m	3	0	82	16	3,908,770	10	6
2K	63	m	3	6	31	27	349,428	10	6
2L	49	m	1	1	33	28	8,254,842	10	12
2M	39	m	3	2	136	33	1,595,253	0	6
2N	53	m	3	nd	26	14	393,794	nd	6
2O	32	m	3	1	615	306	4,481,874	0	6
2P	41	f	3	4	134	64	3,385,406	<15	6
2Q	46	f	5	nd	52	22	7,574,073	632	12
Group 3: treatment success (SVR) with robust CD4^+^ responses									
3A	42	m	3	1	67	48	192,122	0	6
3B	34	f	3	nd	33	32	168,874	10	6
3C	39	m	1	nd	326	24	2,597,732	440	12
3D	54	f	3	3	28	22	507,416	0	6
3E	27	m	1	1	63	27	5,961,828	706	12
3F	67	f	3	1	174	73	223,064	204	6
3G	50	f	1	1	27	23	139,271	10	12

**Figure 1:**
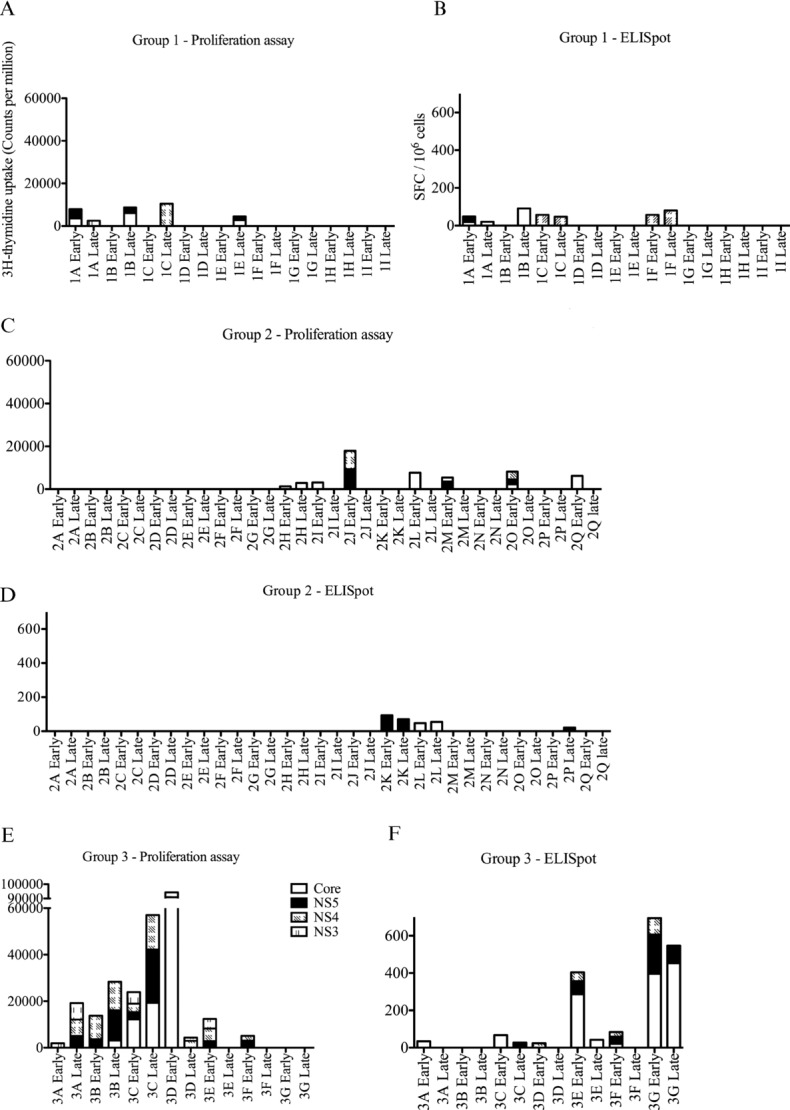
Ex vivo ELISpot (IFN-γ-producing) and cultured T-cell responses in patients undergoing treatment for HCV. Proliferation and ELISpot assays to HCV antigens; core,NS3, NS4, and NS5 were measured at multiple early (days 2, 7, 14, 21, and 28) or late (weeks 12, 24, and 48) time points. (A and B) Group 1 (*n* = 9) shows results for patients who failed treatment. (C and D) Group 2 (*n* = 17) shows patients who successfully cleared virus but with an absent or minimal CD4^+^ T-cell response. (E and F) Group 3 (*n* = 7) shows patients who successfully cleared virus with robust CD4^+^ T-cell responses.

a) Details of patient age and gender are shown as well as the genotype of the infecting HCV. As indicators of hepatic disease, pretreatment liver biopsy modified Knodell fibrosis scores (0–6) are shown alongside ALT levels (normal < 45 IU/ml). Group 1 contains the patients who ultimately failed to clear the virus on treatment; Group 2 contains patients who cleared the virus after treatment (i.e., had an SVR as defined in *Materials and methods*) yet demonstrated minimal CD4^+^ T-cell responses; Group 3 contains patients who cleared the virus after treatment (i.e., had an SVR) and demonstrated robust broad antiviral CD4^+^ T-cell responses. Details of the measured T-cell responses are shown in Fig. 1 and Table 2. No baseline characteristics corresponded to the behavior of the patient immunologically. SVR: sustained virologic response; nd: not determined; NI: necroinflammatory; m: male; f: female.

### Neither ex vivo IFN-γ production nor proliferation of antiviral CD4^+^ T cells correlated with HCV clearance

To determine the role of CD4^+^ T cells in viral clearance, we performed a detailed analysis in the 33 chronically HCV-infected consecutively treated patients whom we had treated for HCV (patient details are shown in Table 1). All 33 patients demonstrated robust responses to the control recall antigens PPD ± TT (where PPD is purified protein derivative and TT is tetanus toxin) in both ex vivo and cultured assays measured at each time point as positive controls (data not shown). We have previously shown that ex vivo responses measure immediate effector type CCR7^−^ CD4^+^ T cells while restimulated cells expanded by in vitro culture reflect a central CCR7^+^ memory type cell [23]. Bearing this in mind and as the largest change in serum viremia occurs early after commencing treatment with IFN-α [24], we considered it important to measure both types of virus-specific CD4^+^ T-cell responses in multiple samples collected during the first month of treatment and at three monthly intervals thereafter. Thus for each individual studied, it was usual to measure at least seven time points. Immune responses were classified as early (measured during the first 28 days) or late (measured from 28 days to 6 months posttreatment).

A patient was recorded as having demonstrated a response to a particular viral protein if two or more results in a period (i.e., early or late) were positive (with at least one proliferation response ≥1000 cpm (counts per minute) above background or at least one ELISpot assay ≥10 antigen-specific spot-forming cells (SFC)/10^6^ PBMCs; as outlined in “Materials and methods”). Patients who failed to obtain an SVR (i.e., treatment failure) were placed in Group 1 (*n* = 9). Patients who gained an SVR (i.e., treatment success) were divided into Group 2 or Group 3 depending on the magnitude of the measured antiviral CD4^+^ T-cell responses. The overall range and duration of early and late ex vivo and cultured antiviral responses are summarized in Table 2 and the actual magnitude is shown in Fig. 1. Group 2 (*n* = 17) identifies subjects who had no detectable responses (seven subjects) or demonstrated a weak transient response (most often to a single protein only) at only two time points in the early or late periods (ten subjects). Hence in Group 2, cumulative proliferation during either early or late periods was <20,000 cpm and cumulative ELISpot during early or late periods was <100 SFC/10^6^ PBMCs. Group 3 (*n* = 7) subjects demonstrated strong robust responses (most often to several different proteins) persistently at multiple time points with cumulative proliferation usually >20,000 cpm or ELISpot > 100 SFC/10^6^ PBMCs during the early and/or late time periods (Fig. 1A–F).

**Table 2:** Summary of the T-cell responses measured by proliferation and ex vivo IFN-γ cytokine productiona)

Patients	Early proliferation	Late proliferation	Early ELISpot	Late ELISpot
Group 1: failed to clear virus after Rx				
1A	+	+	+	+
1B	−	+	−	+
1C	−	+	+	+
1Db)	−	−	−	−
1E	−	+	−	−
1F	−	−	+	+
1Gc)	+	−	−	−
1Hb)	−	−	−	−
1Ib)	−	−	−	−
Group 2: successful treatment outcome — absent or transient CD4^+^ T-cell antiviral responses				
2A	−	−	−	−
2B	−	−	−	−
2C	−	−	−	−
2D	−	−	−	−
2E	−	−	−	−
2F	−	−	−	−
2G	−	−	−	−
2H	−	−	+	−
2I	+	−	+	nt
2J	+	−	−	−
2K	−	−	+	+
2L	+	−	+	+
2M	+	+	−	−
2N	+	−	−	−
2O	+	−	−	−
2P	+	−	−	+
2Q	+	−	−	−
Group 3: successful treatment outcome — persistent/robust CD4^+^ T-cell antiviral responses				
3A	+++	+++	+	−
3B	+++	+++	−	−
3C	+++	+++	+	+
3D	+++	+	−	−
3E	+++	−	++	+
3F	+++	−	−	−
3G	−	−	+++	+++

a) Responses were divided into early (measured at five time points during the first 28 days) or late (measured 4/5 time points for up to 15 months). A subject was considered to demonstrate an early antiviral immune response if assays were positive at any two time points in the first 28 days; a late antiviral response if assays were positive at any two time points after 28 days. For proliferation assays, a positive response to a protein included at least one result >1000 cpm; for an ELISpot assay, a positive response to a protein included at least one result >10 spot forming cells/10^6^ PBMCs.

b) Stopped after 3 months.

c) Stopped after 5 months.

nt: not tested.

+: Transient response to protein (two time points only).

++: Response to one protein at several (>2) time points.

+++: Response to multiple proteins at multiple time points.

−: no response.

The detail of which viral proteins were recognized is summarized in Supporting Information Table 1; no particular viral protein was the focus of ex vivo or cultured responses, although anti-NS3 responses were only found in Group 3. It is clear that a detectable proliferation response is not always associated with an ex vivo cytokine response and vice versa; proliferative and ex vivo responses can focus on the same or distinct proteins. Furthermore, there was no significant difference in the magnitude of antiviral responses to individual viral proteins (Supporting Information Fig. 1). Where responses could be measured, the peak values for proliferative or ex vivo responses did not distinguish between the three groups. Furthermore, it would be impossible to predict treatment outcome based on the presence of CD4^+^ T-cell responses, especially as responses can be demonstrated in patients in Group 1. Twenty-four patients (Groups 2 and 3) cleared the virus and had an SVR. Seventeen patients in Group 2 cleared the virus with minimal/absent measurable CD4^+^ T-cell responses (17/24 or 70%). Indeed the transient nature of responses in this group meant these would have been missed if multiple samples were not taken.

HCV can be grouped into different viral genotypes (1–6; genotypes 1 and 3 are most commonly seen in the United Kingdom). Treatment success is greatest in genotype 3 infection (80%) and poorest in genotype 1 (40%) [25]. In this study, we have found an equal distribution of genotypes across the three groups based on CD4^+^ T-cell responses (Table 1). It is possible for a patient to be infected with a certain HCV genotype (i.e., genotype 1), and spontaneously clear that infection and subsequently be infected with a different genotype virus (such as genotype 3), which establishes a chronic infection [26,27]. Theoretically, such a chronically infected individual may demonstrate robust responses to genotype 1 antigens but not genotype-3-specific responses. In this cohort, there was a marked paucity of responses pretreatment to the antigens used in practically all patients (Supporting Information Fig. 2) and therefore, no evidence of such an effect.

### Increased serum IL-10 does not prevent the clearance of HCV

**Figure 2:**
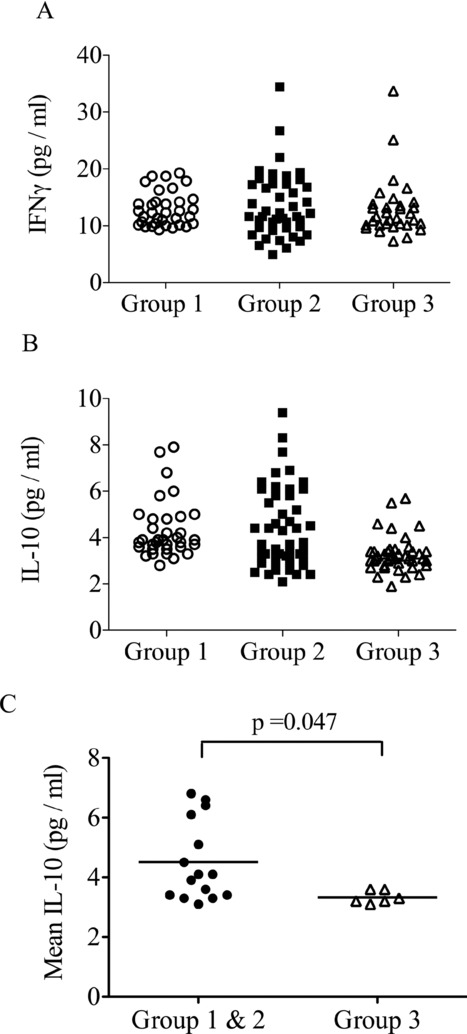
Serum IFN-γ and IL-10 measured over the first 28 days. Serum cytokines were measured at multiple time points over the first 28 days of treatment. Results of multiple samples taken from 21 individuals (*n* = 6, Group 1; *n* = 9, Group 2; *n* = 6, Group 3) of (A) serum IFN-γ and (B) serum IL-10 levels are shown. (C) Mean IL-10 levels for each subject in each group were compared revealing a significant decrease in subjects from Group 3 (*p* = 0.047 unpaired *t*-test).

Multiple serum cytokine levels were measured in these patients from the beginning of treatment. The serum levels of IFN-γ and IL-10 (but not IL-2, IL-4, IL-5, IL-6, and TNF-α: data not shown) were raised above baseline. All measurements made at multiple time points in the first 28 days are shown in Fig. 2A (IFN-γ) and 2B (IL-10). There did not appear to be a difference in the level of IFN-γ between the three groups of HCV patients (Fig. 2A). However, there were lower levels of IL-10 in Group 3 (Fig. 2B). Although IL-10 has been implicated in driving persistence of certain viral infections [28], there were actually significantly higher levels of IL-10 in Group 1 (failed SVR) and Group 2 (SVR, rapid early viral clearance but minimal T-cell responses) compared to Group 3 (SVR with robust T-cell responses; Fig. 2C, *p* = 0.047). The overall association between serum IL-10 and treatment outcome is not clear, but an increased level in some patients in Group 2 does not prevent treatment success.

### Rapid viral clearance is associated with a minimal CD4^+^ T-cell response

**Figure 3:**
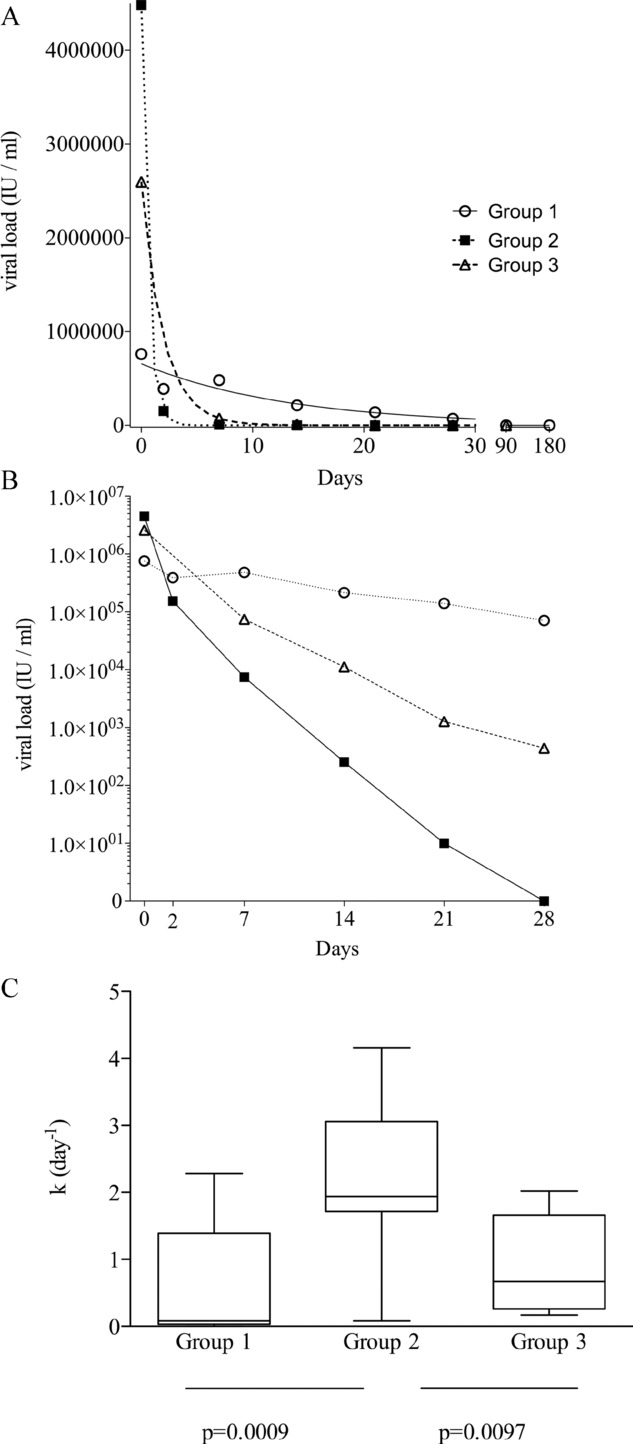
The kinetics of viral clearance. Serum was collected at multiple time points (days 0, 2, 7, 14, 21, 28, and then 3 monthly) and viral load measured. (A) Representative examples of viral clearance are shown for a patient from each of the three groups. *k* (day^−1^) is the rate constant for the viral clearance, the higher value indicating more rapid clearance of virus. The rate constant *k* in day^−1^ of viral clearance is calculated on these curves. Group 1 patient, *k* = 0.08 day^−1^; Group 2, patient *k* = 4.162 day^−1^; Group 3 patient, *k* = 0.51 day^−1^. (B) Viral clearance shown on a logarithmic scale for a representative patient from each group over the first 28 days of treatment. (C) Summary of all the rate constants calculated as in (A) for each group (Group 1, *n* = 9; Group 2, *n* = 17; Group 3, *n* = 7). Data are shown as median (bar), 25th and 75th interquartile (box) and range (whiskers) of the indicated number of samples. The rapid clearance was greatest in Group 2 (*p* = 0.0006, one-way ANOVA).

Dividing the above subjects into three groups on the basis of treatment success or failure, and the magnitude of CD4^+^ T-cell responses, as summarized in Table 2, enabled us to ask further detailed questions regarding other factors influencing the generation of T-cell responses. Viral loads were measured in serum collected and stored from multiple time points (days 0, 2, 7, 14, 21, 28, and then 3 monthly). The changes in viral load after commencing treatment best fitted a nonlinear regression (exponential decline) and the rate of viral clearance for each subject was indicated by the calculated kinetic parameter *k* (day^−1^) as described in *Materials and methods*; representative curves are shown for subjects from Groups 1, 2, and 3 in Fig. 3A and the logarithmic reduction over the first 28 days of treatment is shown in Fig. 3B. There was a highly significant difference (*p* = 0.0006, ANOVA) in the *k* values between the different groups (Fig. 3C): Group 1 — mean *k* 0.60 day^−1^, 95% confidence interval (CI) −0.07 to 1.27; Group 2 — mean *k* 2.25 day^−1^, CI 1.66–2.84; Group 3 — mean *k* 0.92 day^−1^, CI 0.26–1.58). Group 2 demonstrated a significantly more rapid clearance of virus than Groups 1 and 3, but there was no significant difference between Groups 1 and 3. Surprisingly, the most rapid rate of viral clearance after commencing treatment was therefore associated with a paucity of measurable CD4^+^ T-cell responses, and not with the group demonstrating robust CD4^+^ T-cell responses.

### Pretreatment NK-cell phenotype correlated with rate of in vivo viral control in response to IFN-α

**Figure 4:**
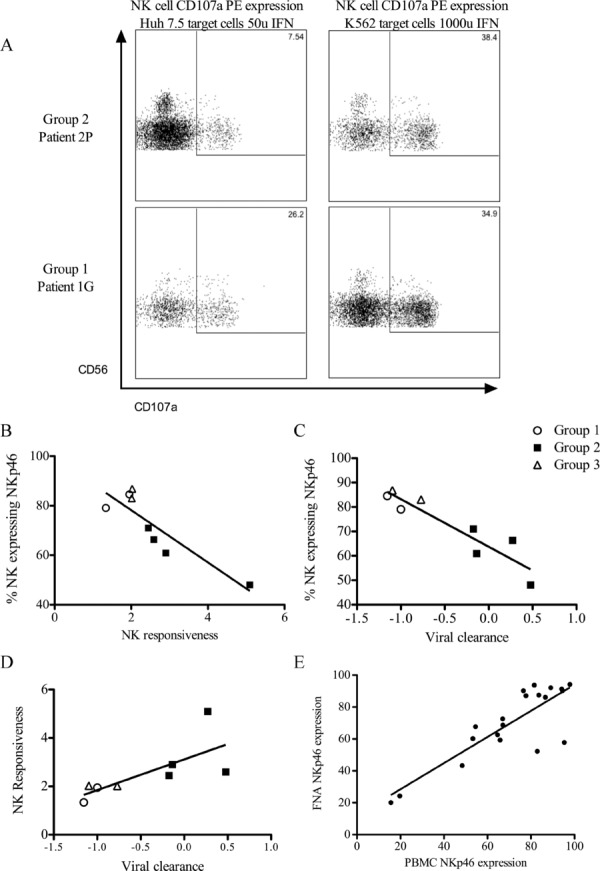
NK-cell phenotype in patients prior to commencing treatment. (A) Representative plots from a patient in Group 2 (top) and Group 1 (bottom) of NK-cell degranulation (CD107a expression) on exposure to a weak stimulus (Huh7.5 cells/50 U IFN-α) and a strong stimulus (K562 cells/1000 U IFN-α). The fold increase in degranulation from a weak to a strong stimulus is far more marked for the Group 3 patient. This was expressed as NK responsiveness = % degranulation with strong stimulus/% degranulation with weak stimulus. Group 2 (top) patient NK responsiveness = 38.4/7.54 = 5.1. Group 1 (bottom) patient = 34.9/28.2 = 1.3. (B) NK-cell NKp46 expression and NK responsiveness (*r*^2^ = 0.78, *p* < 0.004). (C) NKp46 expression and the rate of viral clearance during IFN-α treatment of HCV (*r*^2^ = 0.85, *p* = 0.001). (D) NK responsiveness and the rate of viral clearance (*r*^2^ = 0.51, *p* < 0.05). (E) Peripheral blood NKp46 expression and intrahepatic NKp46 expression (*r*^2^ = 0.7, *p* < 0.0001). These patient samples do not overlap with the treated cohort.

Recently, it has been observed using murine CMV and LCMV that the robustness of the NK-cell response inversely correlates to the development of an adaptive immune response in mice challenged with virus [29–31]. IFN-α-induced NK-cell activation may contribute to the findings above, namely that the rate of control of virus is an important determinant of the type of adaptive CD4^+^ T-cell responses that can develop. We utilized pretreatment frozen samples available in ∼25% (8/33) of the subjects to examine the NK-cell phenotype and responsiveness to type-I IFN. The NK cells were phenotyped using a panel of monoclonal antibodies to recognized NK-cell markers (NKG2D, CD16, NKp30, NKp46; Supporting Information Fig. 3), and their function measured by the ability to degranulate (i.e., circulation of CD107a to and from the cell surface) on activation by type-I IFNs and exposure to target cells. We examined the degree of CD107a surface expression after exposure to a weaker stimulus (Huh7.5 cells plus 50 IU/ml IFN-α) to that with a stronger stimulus (K562 cells plus 1000 IU/ml IFN-α); side by side comparison of NK cells from a patient from Group 1 (failed treatment) with those from a patient from Group 2 (SVR, rapid viral clearance kinetics) is shown in Fig. 4A. In these examples, the weak stimulus had little effect on the NK cells from the patient from Group 2, yet this patient markedly upregulated NK-cell degranulation with the stronger stimulus; this fold increase in NK-cell function with a stronger stimulus was recorded as a ratio, that is, NK responsiveness = % NK cells expressing CD107a with strong stimulus /% NK cells expressing CD107a with a weak stimulus. Thus for this subject with rapid viral clearance, the NK responsiveness was 5.1. This contrasts with the patient from Group 1 who had slower viral clearance and an NK responsiveness of 1.3.

A relationship between baseline NK responsiveness to the rate of viral clearance and baseline expression of the NK-cell marker NKp46 was found. Increased relative ability of NK cells to degranulate in response to a stronger stimulus correlated with low pretreatment NKp46 expression (Fig. 4B, *r*^2^ = 0.78, *p* < 0.004). Low pretreatment NKp46 expression also correlated with the fastest rate of viral clearance (Fig. 4C, *r*^2^ = 0.85, *p* = 0.001). An increased NK responsiveness correlated to cases with a more rapid viral clearance (Fig. 4D, *r*^2^ = 0.52, *p* < 0.05). These figures demonstrate segregation between Group 2 versus Groups 1 and 3. These data support the notion that an increased relative NK responsiveness when comparing a strong to weak stimulus contributes to a more rapid viral clearance, and hence may impinge on adaptive immune responses by control of antigen supply. Although these measurements were made using blood samples, we have subsequently compared the levels of activation markers in blood and intrahepatic NK cells albeit in a different sample population. We found a strong correlation between the levels of NKp46 expression on NK cells derived from blood and corresponding intrahepatic NK cells (Fig. 4E, *p* < 0.0001, *r*^2^ = 0.7), thus indicating that our results using peripheral blood are valid. However, another activating receptor (CD16) had a lower expression in the intrahepatic compartment (Supporting Information Fig. 4), thus demonstrating a distinct intrahepatic NK phenotype and confirming that we were sampling a unique separate compartment. Although other NK-cell markers were measured, in this study we did not find a correlation with NK responsiveness (data not shown).

## Discussion

HCV is an unusual chronic viral infection in humans in as much as treatment can actually clear the virus. Differing rates of control of viremia with treatment enabled interesting human immunological questions to be addressed, such as those pertaining to the relationship and nature of the CD4^+^ T-cell response and the kinetics of antigen clearance. There is very little known in humans regarding the effects of antigen dose on adaptive immune responses [32] and published studies tend to revolve around cytotoxic CD8^+^ T-cell (CTL) responses in HTLV-1 and HIV (reviewed in [33]). As these viruses infect the CD4^+^ T cells, it also confounds analyses of the CD4^+^ T-cell response to antigen.

It is widely accepted that CD4^+^ T-cell responses play a central role in the control of noncytopathic viruses. In HCV infection, we have previously found that patients who cleared the virus demonstrated robust antiviral CD4^+^ T-cell responses [7,8,23] and it seemed reasonable to suppose that CD4^+^ T-cell responses would be more vigorous in subjects who respond to treatment. We measured HCV-specific CD4^+^ T-cell responses not only at multiple time points, but also by both ex vivo IFN-γ production as well as the ability to proliferate. Although cognate T-cell function may be marked by the production of other cytokines such as IL-2 or IL-17, the vast majority of antiviral T cells appear to proliferate and/or produce IFN-γ [34,35], thus it seems unlikely that a useful cognate CD4^+^ T cells response was missed. Given the thoroughness of these measurements, we were surprised that in two thirds (70%) of the prospectively studied patients who successfully cleared HCV (denoted Group 2), there was a paucity or even complete absence of CD4^+^ T-cell responses (Table 2). However, this finding perhaps sheds some light on previous studies which lack a consensus as to whether IFN-α treatment enhances CD4^+^ T-cell function and how CD4^+^ T-cell responses relate to viral control in chronically [5,36–40] and acutely [41–44] infected patients. While spontaneous clearance of HCV is associated with more robust CD4^+^ T-cell responses, it is not clear from the literature whether treatment of chronically infected subjects with IFN-α enhances or diminishes these responses. An earlier study, which examined patients after treatment demonstrated superior responses in the SVR group [38]. However, subsequent studies prospectively measuring responses during treatment have reported conflicting results on the role of CD4^+^ T cells — some reports have categorically stated that there is a poor correlation between CD4^+^ T-cell responses and treatment outcome [36,37,40,42] while others have suggested there is a relationship between rapid viral clearance, SVR and the generation of robust T-cell responses [5,45]. It is noteworthy, particularly when considered alongside our own findings, that those studies suggesting a role for CD4^+^ T cells, usually found this to be the case in only a proportion of patients. Furthermore, recent studies, which have looked at treating acutely infected patients, have suggested that CD4^+^ T-cell responses may be qualitatively altered or even diminished compared to spontaneously resolved infections [43,44].

What emerged as the most striking observation in the patients we studied was the relationship between the kinetics of virus clearance, possibly reflecting antigen clearance, and the presence of antiviral CD4^+^ T-cell responses. Measurement of the rate (*k* in day^−1^) of viral clearance demonstrated that the most rapid viral loss (i.e., higher mean *k*) is actually associated with this absence or poverty of CD4^+^ T-cell responses found in Group 2. Hence, it appears the magnitude of the CD4^+^ T-cell response is dictated by the kinetics of antigen/virus clearance, and not vice versa (Fig. 3C). Rapid loss of virus does not permit a T-cell response to develop, yet the patients go on to clear the virus successfully. Slower rate of antigen loss allowed a response to develop, and in this context, proliferative CD4^+^ T cells may then contribute to the process of viral clearance (Group 3). Serum levels of IL-10 were not different between Group 1 (failed treatment, no SVR) and Group 2, but were significantly lower in Group 3, suggesting a different pathway to viral clearance in this group.

Our results suggest innate, that is, IFN-α-induced, control of virus seemed able to negate and/or prevent the need for a CD4^+^ T-cell response. A recent paper has described how important virus and hence antigen persistence is in IFN-resistant CD169^+^ macrophages in order to facilitate an adaptive immune response after the pathogen has been rapidly cleared by type-I IFNs from other tissues [46]. A similar theme emerges from an observation that has also been described recently using the murine CMV model: strains of mice where NK cells negatively regulate adaptive immune responses do so by the rapid control and removal of antigen (i.e., CMV) [29,31].

NK cells may impinge on adaptive immune responses by altering the function of antigen presenting cells, or even, as suggested recently, by targeting and deleting cognate populations of act-ivated T cells [30,46]. However, in the context of this study, where NK cells could be activated within hours, whereas the measured T-cell responses emerged after approximately a week, it seems most plausible that control of virus/antigen at an early stage may be the most important effect. This reasoning has been corroborated by recent publications implicating early NK-cell activation with treatment outcome [16], and suggestions that these early NK responses reflect differential activation by IFN-α of STAT signaling proteins [47]. Hence, we looked back with available frozen samples at NK-cell function; although we only had samples from 25% of the subjects (*n* = 8; *n* = 2, Group 1; *n* = 4, Group 2; and *n* = 2, Group 3), a significant correlation still emerged. The most rapid rate of viral clearance (and eventual SVR) was associated with a significantly altered pretreatment NK-cell phenotype. These NK cells had a lower level of NKp46 expression and therefore did not respond to the lower level of stimulus. However, these NK cells had a more marked responsiveness as demonstrated by a larger fold increase in cytotoxic function (upregulation of CD107a) when a stronger NK-cell stimulus was employed (Fig. 4). We have previously found an excellent correlation between CD107a expression and IFN-γ production in response to increasing stimulus (TP and AG, unpublished data). Therefore, there is the suggestion that those individuals who have had the greatest increase in cytotoxic response during treatment will also have the greatest increase in IFN-γ production. These results imply that NK cells play a role in antigen control when exposed to IFN-α, and at least supports the emerging belief that rapid antigen removal prevents development of adaptive responses.

Another aspect of this study relates to the impact of IFN-α treatment on antigen-specific CD4^+^ T-cell responses. We recently reported marked augmentation in the responses of memory IFN-γ-producing T cells following exposure to IFN-α with antigen in vitro [12]. However, there does not appear to be an augmentation of preexisting antiviral responses in treated individuals, as the kinetics of the responses when they occurred i.e. developing after the first week, rather resemble a de novo primary response rather than an enhancement of preexisting IFN-γ-producing T cells. Indeed, there is a lack of association of IFN-γ-producing T cells with viral clearance (Table 2).

We have already demonstrated in an individual that HCV can be successfully eradicated with IFN-α without any demonstrable CD4^+^ T-cell or B-cell activity [48]. No clear association has been observed between virus clearance and virus-specific CD8^+^ T-cell activity [42,49]. Thus, it seems that other immune mechanisms must be activated through exposure to high dose IFN-α that are important for virus clearance [50]. The data in this report demonstrate that innate control of virion production and hence the degree of antigen availability seems to be a key step in influencing the development of specific immune responses, and that NK cells may play an important role in shaping adaptive responses. Further studies are required to elucidate precisely the correlates of successful HCV treatment and the full implications of the above findings.

## Materials and methods

### Ethics statement

South East Wales Local Research Ethics Committee (Churchill House, Churchill Way, Cardiff, UK) reviewed this project and granted a favorable ethical opinion for the research (REC reference number 04/WSE03/14 and 10/WSE02/45). Patients who participated gave written consent and ethical committee approval for the study was obtained (Cardiff and Vale Local Ethics Committee, Cardiff and Vale NHS Trust, University Hospital of Wales, Heath Park, Cardiff, UK).

### Patient demographics and clinical samples

It was estimated that all patients recruited had been infected with HCV for >5 years. Thirty-three patients, who went on to complete treatment, were consecutively recruited from clinic over an 18-month period for detailed virological and immunological study (Table 1). Positive anti-HCV antibodies and presence of viral RNA in serum identified by rtPCR confirmed HCV infection. Patients underwent current standard treatment with pegylated-IFN-α and ribavirin for 6–12 months depending on the genotype of virus. Twenty-four patients had a SVR demonstrated by negative PCR tests immediately after treatment and again at 6 months after treatment cessation indicating successful outcome of the HCV therapy. Blood was taken early (days 0, 2, 7, 14, 21, 28) and late (days 90, 180, 360, 540) for analysis of viral loads, serum cytokines, and T-cell responses.

### Antigens

HCV proteins, derived from HCV genotype 1, were purchased from Mikrogen. These include core protein (aa 1–115), nonstructural protein 3 (NS3, aa 1007–1534), NS4 (aa 1617–1864), NS5–4/NS5–12 (NS5, aa 2621–2867/aa 2006–2268). We have previously found HCV genotype 1 proteins will stimulate responses in nongenotype 1 patients [8]. In proliferation and ELISpot assays, antigens were used at a final concentration of 5–10 μg/ml (on the basis of titration curves). These exogenous proteins stimulate CD4^+^ T-cell responses, as shown by CD4^+^/CD8^+^ T-cell depletion experiments [12,23]. Control proteins, tuberculin PPD was purchased from Statens Serum Institut, Denmark; tetanus toxoid protein (TT) was available in house. Both were used at a final concentration of 1 μg/ml.

### Ex vivo cytokine (IFN-γ) ELISpot assays

PBMCs were obtained by density gradient centrifugation and CD4^+^ T-cell responses measured to viral/control antigens by ELISpot assays as previously described [23]. The wells were set up in duplicate. Wells were considered positive if the number of spots was >2× control wells and >5 spots per well; the frequency was calculated after subtracting background and calculated as SFC/10^6^ PBMCs, as described previously [51]. Overall for a patient to be considered as responding to a viral protein, at least one response over the time course had to be >10 SFC/10^6^ PBMCs.

### Proliferation assays

PBMCs were resuspended in RPMI-5% autologous serum at a concentration of 10^6^ cells/ml with viral antigens and antigen-specific proliferation measured by incorporation of ^3^(H) thymidine as previously described [8]. The results were obtained as cpm. The marked decline in background proliferation found when subjects received IFN-α meant the use of the commonly employed stimulation index (ratio of specific proliferation to background proliferation) was not possible due to inflated elevations in the values [12]. Instead, specific proliferation was calculated as mean cpm with antigen minus mean cpm in background control wells. A result was considered positive if the mean antigen driven proliferation was greater than mean background proliferation plus three standard deviations. Overall for a patient to be considered as responding to a viral protein, at least one response over the time course had to be >1000 cpm.

### Cytometric bead array

The quantification of the cytokines (IL-2, IL-4, IL-5, IL-10, TNF-α, IFN-γ) was undertaken using a cytometric bead array (BD Biosciences, Oxford, UK); IL-6 was measured using an in-house ELISA. Ten microliters per sample of each of the capture beads (Th1/Th2) were mixed with serum enhancement buffer and the mixture was then incubated at room temperature in the dark for 30 min with serum before mixing with 50 μl of Human Th1/Th2-II PE detection reagent. The samples (standards and test samples) were incubated in the dark at room temperature for 3 h, washed and analyzed using a BD FACSCalibur and CellQuest Pro software (BD Biosciences).

### Viral load analysis

The concentration of HCV RNA was determined by quantitative RT-PCR (detection down to 15 copies/ml, Cobas Ampliprep/Cobas Taqman HCV test (Roche Diagnostic Systems). This work was undertaken by Lab21 (Cambridge, UK; formerly Delphic Diagnostics, Kent, UK).

### NK-cell assays

Frozen PBMC samples obtained from eight patients prior to treatment were thawed, washed, and reconstituted at 10^6^/ml in RPMI-10% FBS (plus 100 U/ml penicillin, 100 μg/ml streptomycin, 2 mM L-glutamine, 2 mM sodium pyruvate). These cells were plated into 24-well culture plates and cultured over night (16 h) ± 50 IU/ml or 1000 UI/ml of IFN-α (Roferon, Roche Pharmaceuticals). PBMCs were washed and 5 × 10^5^ added to 2×10^5^ K562 (an eyrtholeukaemic cell line lacking MHC class I) or Huh 7.5 (human hepatoma cell line) targets in DMEM-10% FBS. Degranulation assays used fluorochrome-labeled (PE) monoclonal antibody specific to CD107a (BD Biosciences), which was added to the cells and incubated at 37°C for 1 h. Then 1 μl of Golgi stop (BD Biosciences) was added and the cells incubated for a further 3 h. Nonadherent cells were washed and stained with Aqua live/dead stain (Invitrogen, Paisley, UK) and fluorochrome-labeled monoclonal antibodies specific for CD3-APCH7, CD14-APCH7, CD19-APCH7 (BD Biosciences) CD16-FITC (Miltenyi Biotech, Bisley, UK), CD56PerCP.Cy5, and NKp46PB (Biolegend, Cambridge, UK). Cells were then washed and fixed with 2% paraformaldehyde solution. The cells were analyzed by flow cytometry using a CyAn ADP (Beckman-Coulter) and the data analyzed using FlowJo software (Ashland, OR, USA). Degranulation and the expression level of CD107a indicated NK-cell cytotoxicity. We compared the ratio of CD107a upregulation with a strong stimulus (K562 cells plus 1000 IU/ml IFN-α) to a weak stimulus (Huh7.5 cells plus 50 IU/ml IFN-α), that is, NK responsiveness = % NK cells expressing CD107a with strong stimulus/% NK cells expressing CD107a with a weak stimulus.

Intrahepatic NK cells were sampled by fine needle aspiration of the liver from 20 consecutive patients attending for pretreatment liver biopsy. This cohort did not overlap with individuals providing samples for CD4^+^/functional NK data. Samples were stored in RPMI-10% on ice for transport for immediate staining with paired peripheral blood samples. Cells were washed and stained with the antibody panel described above and analyzed by flow cytometry.

### Statistical analysis

In the 33 patients who were studied in detail, outcome was compared to viral load and ALT concentration using Fisher's exact test of proportions. The kinetic parameters of viral clearance (rate constant *k* in day^−1^) were calculated from a nonlinear regression curve (*y* = *y*_0_e^−*kx*^ + plateau; *y*_0_, viral concentration day 0; plateau, final viral concentration after treatment); best fit, that is, one-phase or two-phase exponential decline was decided using 95% CIs and goodness-to-fit calculations (*R*^2^ and absolute sum of squares). Kinetic parameters were compared using a one-way ANOVA and Tukey’s multiple comparison test. Comparison of mean cytokine levels was made using Student’s *t*-test assuming normal distribution of data. To avoid any violation of the independent observations’ assumption, mean levels for each subject were used where multiple samples from each subject were obtained. Comparison of NK degranulation with activation markers was made by linear regression. Software employed was Excel 2003, GraphPad Prism 3, and SPSS 11.

## Abbreviations

ALT: alanine aminotransferase
PPD: purified protein derivative
SFC: spot-forming cell
SVR: sustained virologic response
TT: tetanus toxin

## Notes

Supporting Information available online

**Supplementary Figure 1** The magnitude of proliferative responses to the four HCV antigens is summarized. There was no significant difference in the responses to any particular antigen. **Supplementary Figure 2** Pretreatment CD4^+^ Proliferation and ELISpot responses to antigens. There was a marked paucity of responses. 3 patients had pretreatment responses – 3A 3E and 3G. **Supplementary Figure 3** NK cell gating strategy and surface markers. a) A lymphocyte gate was created based on cell diameter and granularity (side and forward scatter). Single cells were selected and dead cells, CD3^+^ T cells, monocytes and B cells were excluded and CD56^+^ NK cells selected. b) Representative flow cytometry plots for the NK surface markers NKp30, NKp46 and NKG2D prior to commencing treatment. **Supplementary figure 4** Intrahepatic NK cells display a unique phenotype. Compared to the peripheral blood compartment intrahepatic NK cells have reduced CD16 expression [p = 0.0005] but comparable expression of NKp46. (a) representative FACS plot of CD16 expression. (b) CD16 and NKp46 expression in the intrahepatic and peripheral blood compartments. **Supplementary table 1a** HCV antigens recognised in ELISpot assays. **Supplementary table 1b** HCV antigens recognised in proliferation assays.
