# Supplementary material for: Rapid early innate control of hepatitis C virus during IFN-α treatment compromises adaptive CD4+ T-cell immunity
Source: Eur J Immunol. 2012 Aug 6;42(9):2383–94. doi: 10.1002/eji.201142072 (PMC3781703; doi:10.1002/eji.201142072)
Supplement: Supplementary file 1 — Supplementary Figure 1 The magnitude of proliferative responses to the four HCV antigens is summarized. There was no significant difference in the responses to any particular antigen. Supplementary Figure 2 Pretreatment CD4+ Proliferation and ELISpot responses to antigens. There was a marked paucity of responses. 3 patients had pretreatment responses – 3A 3E and 3G. Supplementary Figure 3 NK cell gating strategy and surface markers. a) A lymphocyte gate was created based on cell diameter and granularity (side and forward scatter). Single cells were selected and dead cells, CD3+ T cells, monocytes and B cells were excluded and CD56+ NK cells selected. b) Representative flow cytometry plots for the NK surface markers NKp30, NKp46 and NKG2D prior to commencing treatment. Supplementary figure 4 Intrahepatic NK cells display a unique phenotype. Compared to the peripheral blood compartment intrahepatic NK cells have reduced CD16 expression [p = 0.0005] but comparable expression of NKp46. (a) representative FACS plot of CD16 expression. (b) CD16 and NKp46 expression in the intrahepatic and peripheral blood compartments. Supplementary table 1a HCV antigens recognised in ELISpot assays. Supplementary table 1b HCV antigens recognised in proliferation assays. [file eji0042-2383-sd1.pdf]

European Journal of Immunology

Supporting Information

for

DOI 10.1002/eji.201142072

Tom Pembroke, Ian Rees, Kathleen Gallagher, Emma Jones, Paul Mizen, Timur Navruzov, Andrew Freedman, Ceri Fielding, Ian R. Humphreys, Eddie C. Y. Wang, Awen M. Gallimore and Andrew Godkin

Rapid early innate control of hepatitis C virus during IFN-α treatment compromises adaptive $CD4^{+}$ T-cell immunity

© 2012 WILEY-VCH Verlag GmbH & Co. KGaA, Weinheim

Supplementary Figure 1.

The magnitude of proliferative responses to the four HCV antigens is summarized. There was no significant difference in the responses to any particular antigen.

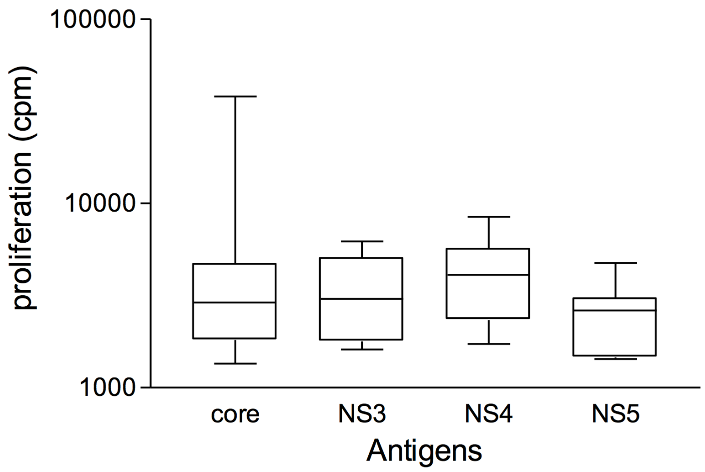

Supplementary Figure 2

Pretreatment CD4$^+$ Proliferation and ELISpot responses to antigens. There was a marked paucity of responses. 3 patients had pretreatment responses – 3A 3E and 3G.

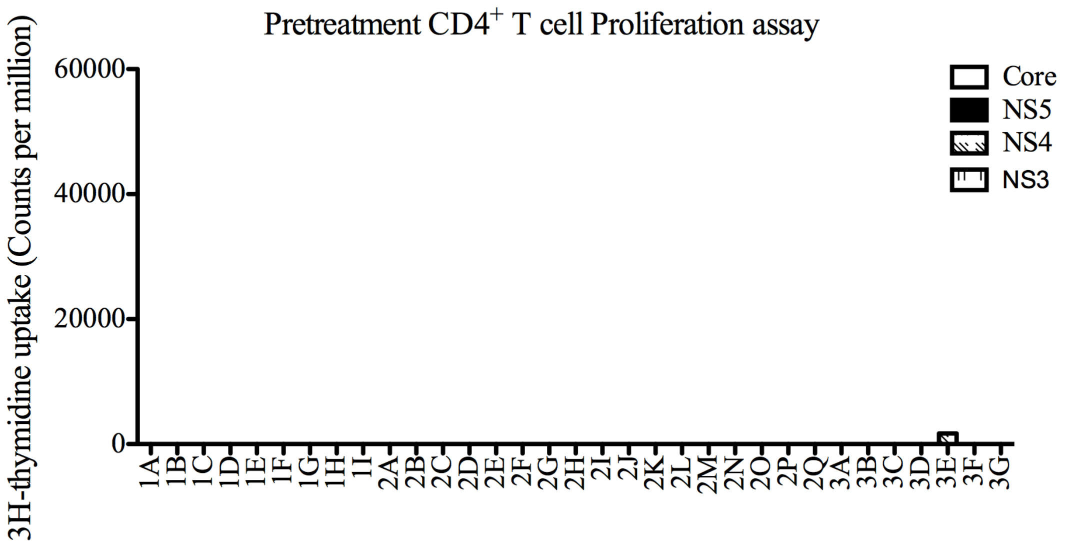

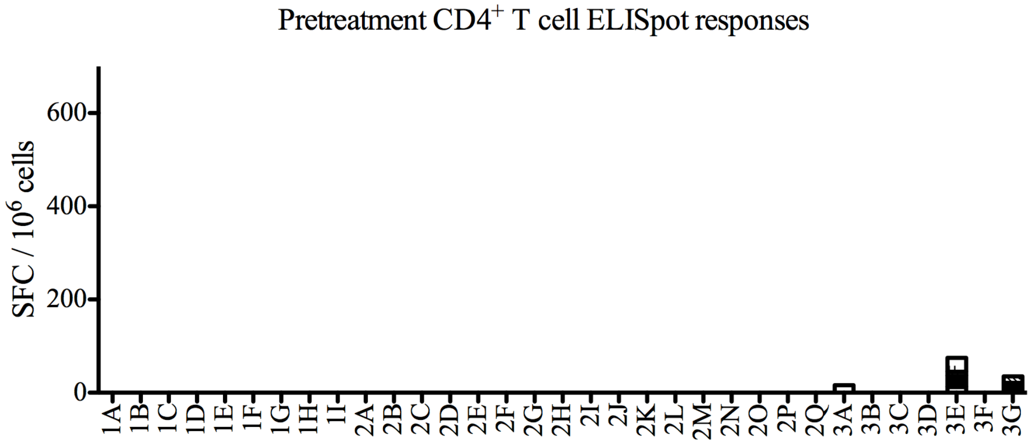

Supplementary Figure 3. NK cell gating strategy and surface markers.

a) A lymphocyte gate was created based on cell diameter and granularity (side and forward scatter). Single cells were selected and dead cells, CD3$^+$ T cells, monocytes and B cells were excluded and CD56$^+$ NK cells selected.

b) Representative flow cytometry plots for the NK surface markers NKp30, NKp46 and NKG2D prior to commencing treatment.

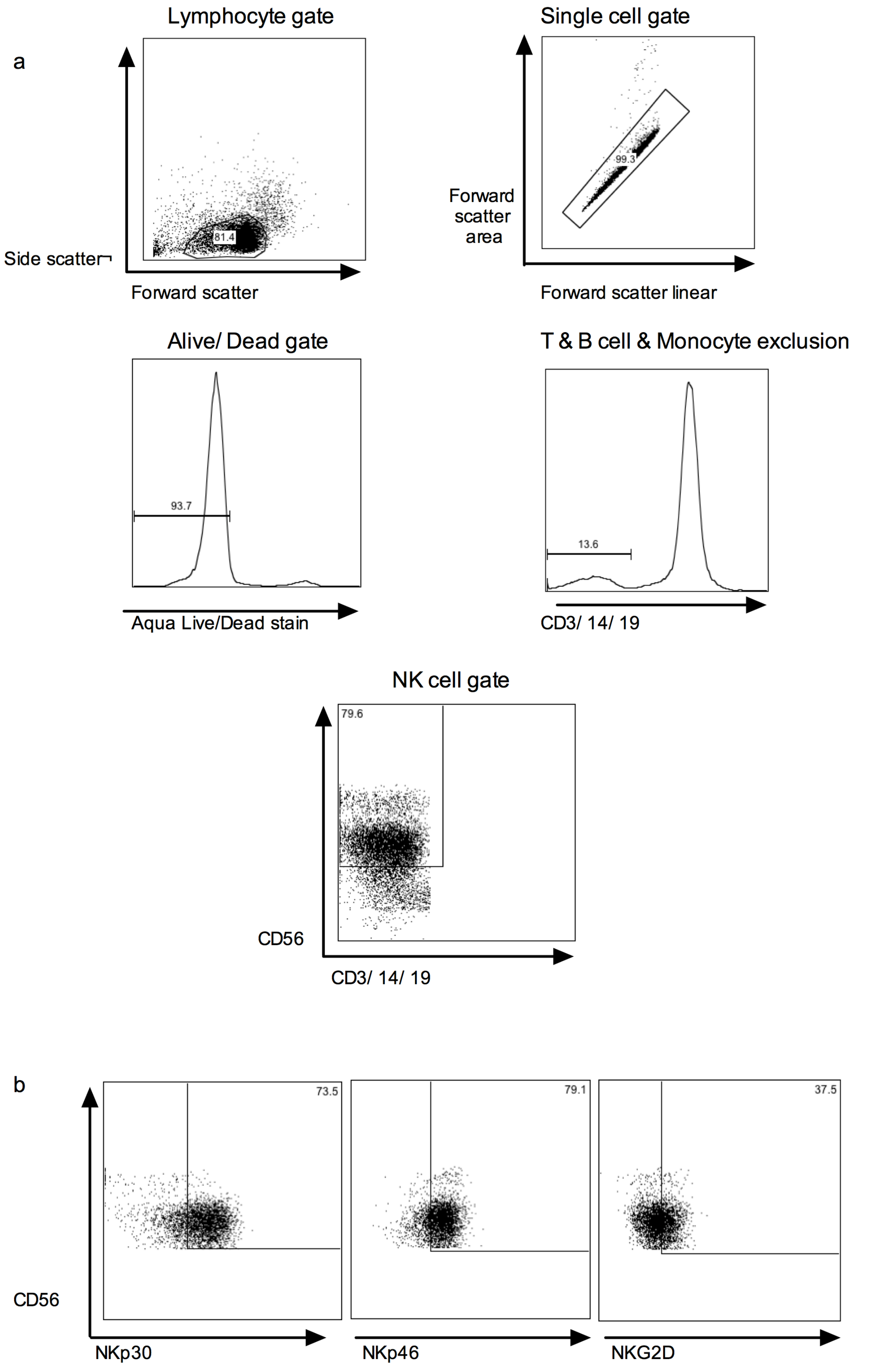

Supplementary figure 4. Intrahepatic NK cells display a unique phenotype.

Compared to the peripheral blood compartment intrahepatic NK cells have reduced CD16 expression [p=0.0005] but comparable expression of NKp46. (a) representative FACS plot of CD16 expression. (b) CD16 and NKp46 expression in the intrahepatic and peripheral blood compartments.

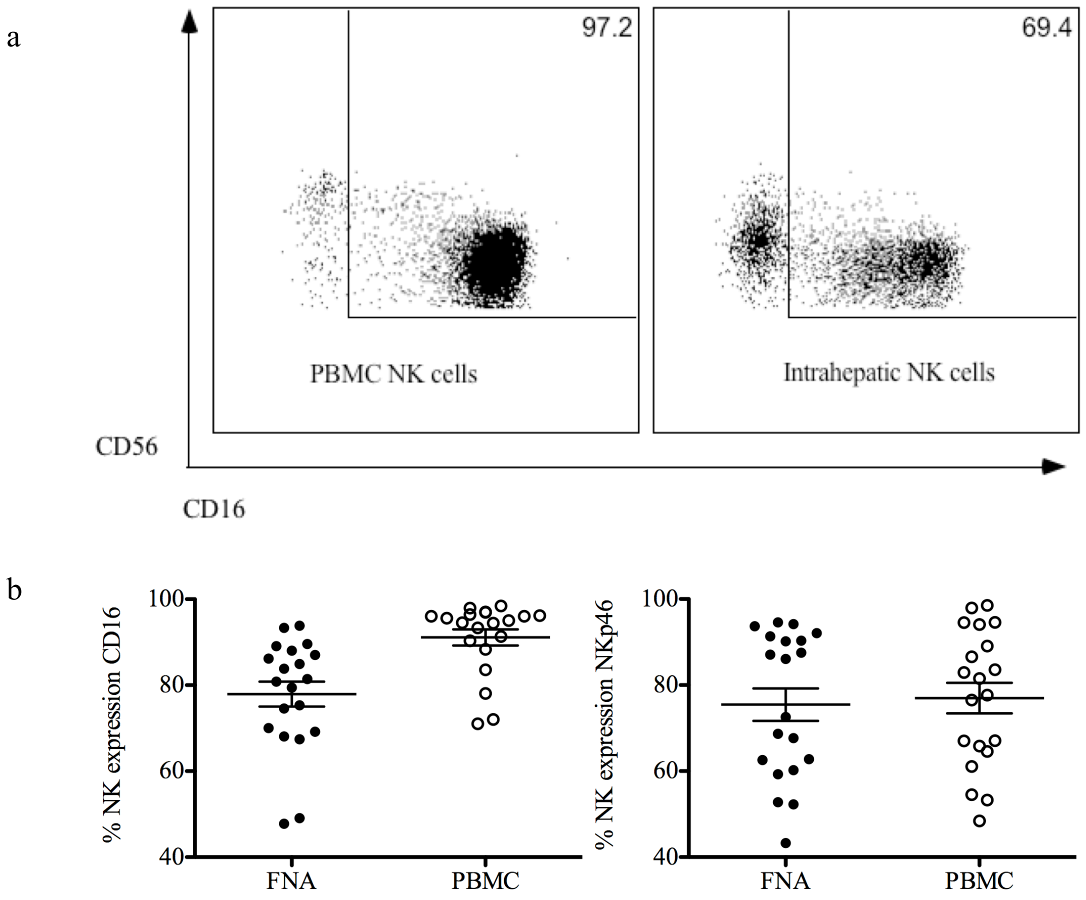

Supplementary table 1a: HCV antigens recognised in ELISpot assays.

**Early = 0 – 28 d**	CORE	NS4	NS5	**Late = 3 – 12 m**	CORE	NS4	NS5
**Group 1: failed to clear virus after treatment**							
1A	+		+	1A			+
1B				1B	+		
1C		+		1C		+	
1D				1D			
1E				1E			
1F		+		1F		+	
1G				1G			
1H				1H			
1I				1I			
**Group 2: successful treatment outcome: absent or transient CD4^{+} T cell anti-viral responses**							
2A				2A			
2B				2B			
2C				2C			
2D				2D			
2E				2E			
2F				2F			
2G				2G			
2H				2H			
					nt	nt	nt
2I				2I			
2J				2J			
2K			+	2K			+
2L	+			2L	+		
2M				2M			
2N				2N			
2O				2O			
2P				2P			+
2Q				2Q			
**Group 3: successful treatment outcome: persistent / robust CD4^{+} T cell anti-viral responses**							
3A	+			3A			
3B				3B			
3C	+			3C			+
3D	+			3D			
3E	+	+	+	3E	+		
3F	+	+	+	3F			
3G	+	+	+	3G	+		+

Supplementary table 1b: HCV antigens recognised in proliferation assays.

	Early = 0 – 28 d					Late = 3 – 12 m			
	CORE	NS3	NS4	NS5		CORE	NS3	NS4	NS5
**Group 1: failed to clear virus after treatment**									
1A			+		1A				+
1B					1B	+			
1C					1C			+	
1D					1D				
1E					1E	+			+
1F					1F				
1G		+	+	+	1G				
1H					1H				
1I					1I				
**Group 2: successful treatment outcome: absent or transient CD4$^+$ T cell anti-viral responses**									
2A					2A				
2B					2B				
2C					2C				
2D					2D				
2E				+	2E				
2F					2F				
2G					2G				
2H				+	2H				
	+								
2I					2I				
2J					2J				
2K					2K				
2L	+				2L				
2M	+				2M	+		+	
2N					2N				
2O	+			+	2O				
2P					2P				
2Q	+				2Q				
**Group 3: successful treatment outcome: persistent / robust CD4$^+$ T cell anti-viral responses**									
3A		+			3A		+	+	+
3B			+	+	3B			+	+
3C	+	+	+	+	3C	+		+	+
3D	+	+	+	+	3D	+			
3E	+	+	+	+	3E				
3F	+	+	+	+	3F				
3G					3G				

Summary of antigens recognised by viral specific CD4+ T cells by (A) *ex vivo* IFNγ-producing or by (B) proliferation after 6 days culture. The immune response was directed to all viral antigens, although NS3 was favoured in group 3. The only striking difference in the type of response between patients who fail to eradicate the virus on treatment (Group 1) vs. the successfully treated patients (Groups 2 and 3) is in early proliferative responses (Group 1 vs. Group 3: $p = 1.4 \times 10^{-9}$, Group 1 vs. Groups 2: $p = 0.0018$, chi squared test).
